# Validation of PROMIS anxiety item bank computer adaptive test among patients with heart failure

**DOI:** 10.3389/fcvm.2025.1605130

**Published:** 2025-10-30

**Authors:** Nolan Marblestone, Steven Chu, Nicole Tomei, Denzel Lodge, Aarushi Bansal, Nathaniel Edwards, Heather J. Ross, Josef Stehlik, Desana Thayaparan, Jad Fadlallah, Joshua G. Lee, Istvan Mucsi

**Affiliations:** ^1^ Ajmera Transplant Centre and Division of Nephrology, University Health Network, Toronto, ON, Canada; ^2^ Ted Rogers Centre for Heart Research (TRCHR), Toronto, ON, Canada; ^3^ ICES, Institute for Clinical Evaluative Services, Toronto, ON, Canada; ^4^ Department of Medicine, Division of Cardiology, McMaster University, Hamilton, ON, Canada; ^5^ Peter Munk Cardiac Centre, University Health Network, Toronto, ON, Canada; ^6^ Division of Cardiovascular Medicine, University of Utah, Salt Lake City, UT, United States; ^7^ Department of Medical Sciences, Western University, London, ON, Canada

**Keywords:** PROMIS (Patient-Reported Outcomes Measurement Information System), PROMS (patient-reported outcome measures), anxiety, heart failure, validation, mental health

## Abstract

**Introduction:**

Anxiety is highly prevalent among patients with heart failure (HF), negatively affecting health related quality of life (HRQOL). The Patient-Reported Outcomes Measurement Information System (PROMIS) anxiety item bank computer adaptive testing (CAT) precisely assesses anxiety symptom severity. This study aims to assess construct validity and reliability of PROMIS-Anxiety CAT among patients hospitalized for HF.

**Methods:**

A cross-sectional convenience sample of adult patients hospitalized for HF, who completed PROMIS-A CAT, generalized anxiety disorder 7 (GAD-7), and other questionnaires electronically. Convergent validity was assessed by Spearman's rank correlation between PROMIS-A CAT, GAD-7, and other legacy measures. Known group analysis compared PROMIS-A CAT and GAD-7 scores between groups expected to have different levels of anxiety. Reliability of PROMIS-A CAT was calculated on the individual and group level from standard error of measurement, according to item response theory. Area under receiver-operating characteristics (ROC) curve and Youden's J statistic were used to identify a *T*-score cut-off for moderate/severe anxiety.

**Results:**

Of 333 participants, 87 (26%) had moderate/severe anxiety based on GAD-7 score (≥ 10). Participants completed on average (median [IQR]) 4(1) vs. 7(0) items, with PROMIS-A CAT and GAD-7, respectively. PROMIS-A CAT *T*-scores were strongly correlated with GAD-7 scores (rho = 0.78) and moderately correlated with other legacy measures. Known-group analysis provided further support for construct validity of PROMIS-A CAT. Individual reliability for PROMIS-A CAT *T*-scores was >0.9 for 87% of the sample; mean reliability was 0.91. Based on ROC and Youden's J analyses, a *T*-score of 60 can be used to identify individuals with moderate/severe anxiety.

**Conclusion:**

These results support the validity and reliability of PROMIS-A CAT among patients hospitalized for HF.

## 1 Introduction

Approximately 750,000 Canadians live with heart failure (HF), with about 100,000 new patients diagnosed annually (1). HF is characterized by signs and symptoms of congestion (i.e., shortness of breath, orthopnea, jugular venous distention, and pedal edema) that result from structural and/or functional cardiac abnormalities causing elevated cardiac pressures and reduced cardiac output (2). Patients with HF may experience diverse psychological and physical symptoms, which can contribute to impaired health-related quality of life (HRQOL) and increased healthcare utilization (3, 4).

Reportedly, 29%–53% of patients with HF have clinically relevant anxiety symptoms (3, 5–7). Anxiety is frequently underdiagnosed and undertreated in patients with chronic medical conditions, including HF (8, 9). Treating anxiety among patients with HF may improve outcomes, underscoring the importance of early screening, diagnosis, and treatment (8).

Patient-reported outcomes (PROs) are reports directly from patients regarding their functional abilities, symptoms, and feelings related to a health condition and its treatment (10). Patient-reported outcomes measures (PROMs) are standard questionnaires used to measure PROs, including anxiety (11). The use of PROMs when linked to appropriate symptom management pathways can improve clinical outcomes, quality of care, and communication between patients and healthcare providers (12–14).

The Generalized Anxiety Disorder 7 (GAD-7) questionnaire is a 7-item tool that is widely used to assess anxiety symptoms (15). However, tools like GAD-7 have been developed based on Classical Test Theory. These instruments include items that cover the whole symptom severity spectrum (including both the high and the low end), requiring all or most items to be completed by participants to obtain valid and reliable scores. Consequently, respondents may be obliged to complete irrelevant items, which can lead to high questionnaire burden, respondent fatigue, poor completion rates, and compromised data quality (16, 17).

The Patient-Reported Outcomes Measurement Information System (PROMIS) developed and validated item banks to measure generic, clinically actionable PROs, relevant across various medical conditions (18). PROMIS item banks have been developed using the Item Response Theory (19, 20), where each item and every response option is calibrated to a *T*-score. Consequently, any combination from the item bank can be used, depending on the specific context. PROMIS and other tools developed by IRT can be administered as fixed-length short forms (SF); 2–4 item SF are typically the shortest options, with 4–10 item SF available as well. An alternative administration method is computer adaptive testing (CAT) (21). When administering an item bank via CAT, all participants answer an initial item that is calibrated for average symptom severity; subsequent items are selected by an algorithm based on prior responses, ensuring each question is relevant to an individual's symptom severity or level of functioning (22, 23). Items are delivered until a stopping rule is met; often when reliability over 90% is reached or 12 items are completed (24). CAT therefore delivers tailored questionnaires, omitting irrelevant items while maintaining high precision, which may increase completion and adherence rates.

PROMIS instruments, therefore, offer excellent measurement precision with tailored questions to reduce question burden. Other studies have confirmed good measurement characteristics of the PROMIS-A CAT, reporting high reliability and good construct validity among multiple patient populations including those with chronic kidney disease and chronic pain (25, 26). Additionally, there is evidence supporting the feasibility of routine PROMIS-A CAT use in an in-patient setting (27). However, further analysis is required to ensure these measurement characteristics remain acceptable among diverse patient populations and to set population-specific, clinically actionable thresholds. This is the first study to assess the validity and reliability of PROMIS Anxiety Computer Adaptive Test (PROMIS-A CAT) in measuring anxiety symptoms among hospitalized patients with HF.

## 2 Methods

### 2.1 Study design & patient population

This analysis was completed with a cross-sectional, convenience sub-cohort of adult (≥18 years) patients experiencing HF, who were enrolled in the “*Predicting Readmission Outcomes using Biostatistical Evaluation and Machine Learning (PROBE ML)*” study at Toronto General Hospital and Toronto Western Hospital between March 2019 and October 2022. Patients were considered for inclusion in the study if they were admitted with a diagnosis of HF. Clinical diagnosis of HF was guided by the Framingham criteria for HF and/or serum BNP levels >100 pg/ml (28). Serum BNP was measured with a two-step chemiluminescent assay using the Abbott Architect i2000 analyzer. Patients were excluded (exclusion criteria were informed by the objectives of the *PROBE ML* study) if they were diagnosed with dementia, had severe cognitive deficits, underwent a heart or double lung transplant, had active cancer, did not speak English, were not Ontario residents, or were currently undergoing dialysis. Patients with a “Do Not Resuscitate” (DNR) order or those receiving end-of-life palliative care were also excluded. Similarly, patients who lived in long-term care or nursing home facilities, or were scheduled for discharge to such facility, were not enrolled as these patients were expected to require a different level of care and experience higher readmission rates (29). For this analysis, those who did not complete legacy questionnaires were also excluded. All participants provided written informed consent before enrolment. Research Ethics Board approval was obtained (REB#18-5658).

### 2.2 Questionnaire administration

Patients were approached by research team members during admission and invited to participate in the *PROBE ML* study. Approximately the first 300 consenting participants were administered both PROMIS and legacy measures (established, valid questionnaires measuring the specific construct of interest) for validation of the PROMIS tools; this sample gives over 90% power to detect the target correlation (rho = 0.6) at an alpha of 0.05. These participants completed multiple PROMIS domains assessed via CAT (PROMIS Bank v1.0: anxiety, fatigue, depression, dyspnea severity; PROMIS Bank v2.0: physical function, PROMIS Global Health 10 v1.1) and legacy questionnaires using a tablet-based electronic data capture (Data Driven Outcomes System, TECHNA Institute, University Health Network, Toronto).

### 2.3 Sociodemographic and clinical characteristics

Sociodemographic characteristics were obtained by trained chart abstractors from the Institute for Clinical Evaluative Sciences (ICES). Clinical laboratory variables were obtained from a blood sample (15 cc for plasma, serum, and buffy coat), collected within one day of study enrolment for the majority (90%) of participants. From these samples, hemoglobin, serum creatinine, serum sodium, and serum BNP level were measured. HF characteristics included etiology and HF type, classified as follows: HF with reduced ejection fraction (HFrEF) [left ventricular ejection fraction (LVEF) <40%]; HF with mildly reduced ejection fraction (HFmEF) (LVEF 40%–49%); or HF with preserved ejection fraction (HFpEF) (LVEF ≥50%). HF characteristics, etiology, and comorbidities [to calculate the Charlson Comorbidity Index (CCI)] were obtained from medical records.

### 2.4 PROMIS-anxiety item bank

The PROMIS Anxiety item bank v1.0 for adults includes 29 items that assess fear, anxious state, hyperarousal, and somatic experiences related to mental arousal. Each item requires patients to rate frequency of particular events on a 5-point Likert scale (“never”, “rarely”, “sometimes”, “often”, and “always”), where higher scores correspond to greater levels of anxiety (30). Raw scores are converted to a standardized *T*-score, where a mean score of 50 and standard deviation (SD) of 10 corresponds to the mean (SD) anxiety score of the United States general population (18). When administered via CAT, participants complete a minimum of 4 items. Items are administered until a standard error of measurement (SEM) of <0.3 (reliability >0.90) is achieved or participants have completed 12 items, according to the established stopping rule (24, 31, 32).

### 2.5 Legacy questionnaires

Construct validity of PROMIS-A CAT was assessed by analyzing correlations between PROMIS-A CAT *T*-scores and legacy questionnaire scores. The Generalized Anxiety Disorder-7 (GAD-7) was selected as the primary legacy instrument. This 7-item questionnaire assesses anxiety symptoms based on the Diagnostic and Statistical Manual of Mental Disorders, Fifth Edition criteria for generalized anxiety (33). It uses a 4-point Likert scale ranging from 0 (“not at all bothered”) to 3 (“bothered nearly every day”) to measure severity of self-reported anxiety, with total scores ranging from 0 to 21. Scores ≥10 indicate moderate to severe anxiety symptoms (15).

The Edmonton Symptom Assessment System-revised (ESAS-r) measures the severity of nine emotional and physical symptoms on an 11-point scale, ranging from 0 (“no”) to 10 (“worst possible”) (34, 35). The anxiety item from this tool was used as the secondary legacy instrument for this analysis. The ESAS-r has demonstrated reliability and validity in patients with HF (36).

The EuroQol 5-Dimension 5-Level (EQ-5D-5l) assesses self-rated health over 5 domains: mobility, self-care, usual activities, pain/discomfort, and anxiety/depression (37, 38). The EQ-5D-Anxiety/Depression item was used as the tertiary legacy instrument for this analysis. It uses a 5-point scale, ranging from “not anxious or depressed” to “I am extremely anxious or depressed” (39). This questionnaire has documented reliability and validity among patients with heart disease (40).

The Patient Health Questionnaire-9 (PHQ-9) is a 9-item measure of depression, with each item assessed on a 4-point scale from “not at all” to “nearly every day”. Scores range from 0 to 27, with scores ≥10 representing moderate depression severity (41).

The Kansas City Cardiomyopathy Questionnaire-12 (KCCQ-12) is a 12-item, self-administered tool that assesses HRQOL in patients with HF. It consists of four domains: physical limitation, symptom frequency, quality of life, and social limitations; all are individually scored from 0 (worst possible health) to 100 (best possible health). The KCCQ-12 summary score is calculated as the mean of the four subdomain scores (42).

### 2.6 Statistical analysis

Baseline descriptive statistics are presented as mean (SD) for normally distributed variables, median [interquartile range (IQR)] for skewed variables, and frequency (%) for categorical variables. Characteristics between participants with vs. without moderate/severe anxiety (cut-off score: GAD-7 ≥10) were compared using independent sample *T*-tests for normally distributed variables and Mann–Whitney *U*-tests for nonparametric variables. Normality was assessed by a visual inspection of a density plot and QQ-plot for all variables, as well as Pearson's coefficient of skewness for PROMs scores. Categorical variables were compared using chi-squared tests. Bonferroni correction for multiple tests was used to determine a significant alpha threshold by dividing an alpha of 0.05 by number of tests performed (43). Floor and ceiling effects were calculated as the percentage of participants who scored at the minimum and maximum possible questionnaire score, respectively. Skewness of PROM scores was quantified using Pearson's moment coefficient of skewness. A coefficient of 0 represents a symmetric distribution (i.e., normal distribution); a large positive coefficient indicates a right-skewed distribution, whereas a large negative coefficient indicates left-skewness (44).

The reliability of the PROMIS-A CAT was assessed at both the individual and group level. We calculated individual-level reliability from SEMs across the PROMIS-A CAT score spectrum using the formula: reliability = 1 − SEM^2^ to obtain values ranging from 0 (no reliability) to 1 (perfect reliability). Reliability ≥0.90 (SEM = 0.32), is considered acceptable for an individual score (45). Group level reliability was calculated using the formula: average reliability = 1 − [mean(SEM)]^2^, where reliability ≥0.90 is considered acceptable (31). Cronbach's alpha was calculated to assess internal consistency of the GAD-7, ESAS-r, and EQ-5D-5l. Alpha values between 0.80 and 0.89 indicate good internal consistency, while values >0.90 indicate excellent internal consistency (46).

Convergent validity was assessed by examining *T*-score correlations between PROMIS-A CAT and legacy measures assessing the same or similar construct (GAD-7, ESAS-r Anxiety, and EQ-5D-Anxiety/Depression). Moderate correlation (rho 0.5–0.7) is considered acceptable, while a strong correlation (rho >0.7) shows excellent validity (47, 48). Divergent (discriminant) validity was assessed by examining *T*-score correlations between PROMIS-A CAT and tools measuring constructs unrelated to anxiety (ESAS-r Appetite, KCCQ-Physical Limitation, and the EQ-5D-Mobility). We expected weak correlations (rho < 0.4) between these measures (49).

To further analyze construct validity, we compared mean PROMIS-A CAT *T*-scores and median GAD-7 scores between groups expected to have different levels of anxiety. We expected higher anxiety among participants who were female (50), were younger (51), had HFrEF (52), and had greater comorbidity (53). We established additional groups based on legacy PROMs. We used the ESAS-r symptom score cut-off ≥30 to define high global symptom burden and expected these participants to have higher anxiety (23). We used a PHQ-9 score (cut-off ≥ 10) to define moderate to severe depressive symptoms and expected these participants to have higher anxiety (41, 54). Lastly, we used the KCCQ-12 summary score (cut-off ≥ 25) to indicate good HRQOL, expecting participants with impaired HRQOL (<25) to have higher average anxiety (55). For the known group analyses, PROMIS-A CAT *T*-scores and GAD-7 scores were adjusted for age, sex, and EF. Adjusted PROMIS-A CAT *T*-scores were obtained from linear regression model least-squared means. Adjusted median GAD-7 scores were obtained from quantile regression predicted medians. Group comparisons of adjusted mean PROMIS-A CAT *T*-scores and adjusted median GAD-7 scores were conducted using independent sample *T*-tests for binary group PROMIS-A CAT *T*-scores, Mann–Whitney *U*-tests for binary group GAD-7 scores, analysis of variance (ANOVA) for PROMIS-A CAT *T*-scores with more than 2 groups, and Kruskal–Wallis tests for GAD-7 scores with more than 2 groups. Cohen's D effect size was calculated for all binary comparisons of PROMIS-A CAT *T*-scores, which was classified as classified as 0–0.49 (small), 0.5–0.79 (moderate), and >0.8 (large) (56). To account for the skewed distribution of GAD-7 scores, bootstrap resampling with 1000 replication was used to estimate Cliff's delta effect size—classified as 0–0.32 (small), 0.33–0.46 (moderate) >0.47 (large) (57).

To assess discrimination of the PROMIS-A CAT, we conducted receiver operating characteristics (ROC) analysis using a GAD-7 score ≥10 (indicating moderate to severe anxiety) as the reference (15, 58). Test discrimination was measured by the area under the ROC curve (AUROC), with 0.7–0.8, 0.8–0.9, and >0.9 representing acceptable, excellent, and outstanding discrimination, respectively (59–61). Youden's *J* index was used to identify a clinically relevant cut-off score for the PROMIS-A CAT to identify HF patients with moderate to severe anxiety symptoms.

Missing data were not imputed as fewer than 5% of participants were missing any observations used in multivariate adjustment for known-groups comparison. Statistical analyses were performed using Stata version 15.1 and R version 4.3.3.

## 3 Results

Among the 520 patients enrolled in the main study, 333 completed both PROMIS-A CAT and legacy instruments for validation. Participant characteristics are presented in Table 1. Mean (SD) age was 67 (16) years with 217 (65%) male participants. Of these participants, 87 (26%) had moderate to severe anxiety (GAD-7 score ≥ 10). The most common etiology of HF in this cohort was non-ischemic cardiomyopathy, affecting 218 (66%) participants; those with non-ischemic cardiomyopathy made up a higher proportion of the moderate to severe anxiety cohort (77% vs. 62%, *p* = 0.013). Those with moderate to severe anxiety were younger [mean (SD) age 61 (16) vs. 70 (15) years, *p* < 0.001].

**Table 1:** Participant characteristics.

Characteristics	Total (*n* = 333)	Patients without moderate/severe anxiety (GAD-7 < 10) (*n* = 246)	Patients with moderate/severe anxiety (GAD-7 ≥ 10) (*n* = 87)	*P*-value^a^
Age (years); mean (SD)	67 (16)	70 (15)	61 (16)	<0.001
Sex (male); *n* (%)	217 (65)	166 (67)	51 (59)	0.14
Marital status; *n* (%)				0.7
Single	19 (6)	14 (6)	5 (6)	
Married or common law	153 (46)	117 (48)	36 (41)	
Divorced, separated, widowed	14 (4.2)	11 (4.5)	3 (3.4)	
Education; *n* (%)				0.30
<12 years	39 (13)	32 (15)	7 (9)	
≥12 years	255 (87)	188 (85)	67 (91)	
Smoking (current/former); *n* (%)	102 (31)	71 (29)	31 (36)	0.2
Serum creatinine (umol/L); mean (SD)	138 (69)	137 (65)	142 (79)	0.9
Blood hemoglobin (g/L); mean (SD)	120 (23)	120 (23)	121 (22)	>0.9
Serum sodium (mmol/L); mean (SD)	137 (5)	137 (4.6)	138 (4.2)	0.3
Serum BNP Level (pg/ml); mean (SD)	1,307 (1,098)	1,265 (1,029)	1,428 (1,278)	0.6
Charlson comorbidity index; *n* (%)				0.9
<4	288 (86)	213 (87)	75 (86)	
≥4	45 (14)	33 (13)	12 (14)	
LVEF; mean (SD)	37 (17)	37 (17)	35 (17)	0.3
HF type; *n* (%)				0.6
HFrEF (LVEF <40%)	171 (54)	124 (53)	47 (56)	
HFmEF (LVEF 40%–49%)	47 (15)	33 (14)	14 (17)	
HFpEF (LVEF ≥50%)	101 (32)	78 (33)	23 (27)	
CHF etiology^b^; *n* (%)				
CAD/Ischemia	105 (32)	88 (36)	17 (20)	0.006
Valvular	47 (14)	36 (15)	11 (13)	0.7
Hypertension	8 (2.4)	6 (2.4)	2 (2.3)	>0.9
Non-ischemic cardiomyopathy	218 (66)	152 (62)	66 (77)	0.013

GAD-7, General Anxiety Disorder-7; SD, standard deviation; IQR, interquartile range; BNP, B-type natriuretic peptide; LVEF, left ventricular ejection fraction; HFrEF. heart failure with reduced ejection fraction; HFmEF, heart failure with mid-range ejection fraction; HFpEF, heart failure with preserved ejection fraction; HF, heart failure; CHF, congestive heart failure; CAD, coronary artery disease.

^a^ Alpha threshold for significance after Bonferroni correction for multiple tests = 0.003.

^b^ CHF etiologies are not mutually exclusive.

Summary statistics for PROMIS and legacy measures are presented in Table 2. The median (IQR) GAD-7 score was 5 (8), with a higher floor effect (7%) and skewness (0.88) compared to PROMIS-A CAT *T*-scores [median (IQR) 56 (16), floor effect (5%), skewness (−0.19)] (Supplementary Figure S2). The median (IQR) number of PROMIS-A CAT items completed was 4 (1), with a range of 4–12. Seventy-five percent of participants completed ≤5 PROMIS-A CAT items.

**Table 2:** Summary statistics of PROM scores.

PROM	Mean (SD)	Median (IQR)	Cohort score range	Floor effect (%)	Ceiling effect (%)
PROMIS-anxiety *T*-score v.1.0	55 (11)	56 (16)	33–85	5	1
GAD-7	6 (6)	5 (8)	0–21	17	2
ESAS-r anxiety	3 (3)	1 (5)	0–10	43	2
ESAS-r appetite	3 (3)	1 (5)	0–10	48	3
EQ-5D-5l anxiety/depression	2 (1)	2 (3)	1–5	46	2
PHQ-9	9 (6)	9 (9)	0–27	5	1
KCCQ-12 physical limitation	42 (29)	42 (40)	0 −100	11	5
KCCQ-12 summary score	36 (24)	33 (34)	0–98	1	0

PROM, patient-reported outcome measure; SD, standard deviation; IQR, interquartile range; PROMIS, Patient-Reported Outcome Measurement Information System; GAD-7, Generalized Anxiety Disorder-7; ESAS-r, Edmonton Symptom Assessment System Revised; EQ-5D, EuroQol 5-Dimension 5-Level; PHQ-9, Patient-Health Questionnaire-9; KCCQ-12, Kansas City Cardiomyopathy Questionnaire-12.

**Figure 1:**
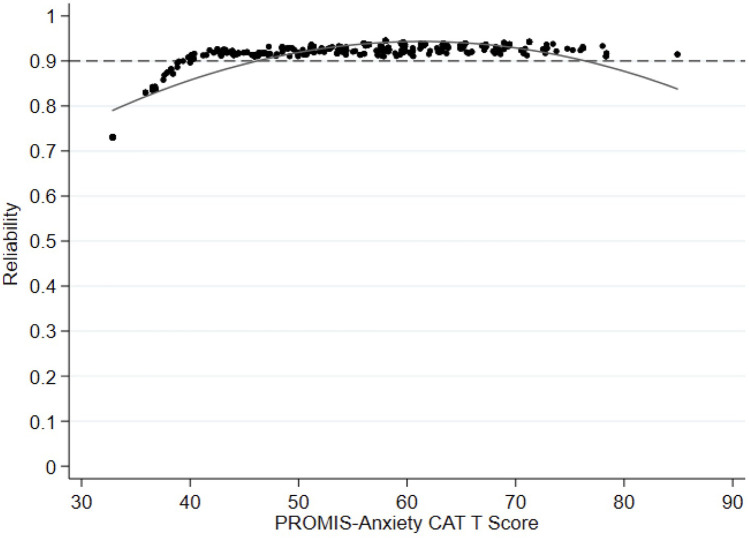
Reliability plot comparing anxiety levels (PROMIS-anxiety CAT T score theta) with reliability (=1 − [mean(SME^2^)] the entire cohort. PROMIS, Painted-Reported Income Measurement Information System; CAT, computer Adaptive Test.

The mean reliability of PROMIS-A CAT for the total sample was 0.91, with reliability above 0.9 for 87% of participants (see Figure 1 for reliability across all *T*-scores). Cronbach's alpha was 0.9 [95% confidence interval (CI): 0.89–0.92] for the GAD-7, 0.8 (95% CI: 0.82–0.87) for the ESAS-r, and 0.78 (95% CI: 0.75–0.81) for the EQ-5D-5l.

PROMIS-A CAT *T*-scores were strongly correlated with GAD-7 scores (rho = 0.78, 95% CI: 0.73–0.82, *p* < 0.001) and moderately correlated with ESAS-r Anxiety (rho = 0.62, 95% CI: 0.55–0.70, *p* < 0.001) and EQ-5D-Anxiety/Depression (rho = 0.67, 95% CI: 0.60–0.73, *p* < 0.001), as expected (Table 3). PROMIS-A CAT *T*-scores correlated only weakly with constructs unrelated to anxiety, including ESAS-r Appetite (rho = 0.18, 95% CI: 0.07–0.28, *P* < 0.001), EQ-5D-Mobility (rho = 0.20, 95% CI: 0.10–0.31, *p* < 0.001), and KCCQ-Physical limitation (rho = −0.27, 95% CI: −0.38 to −0.16, *p* < 0.001) (Table 3).

**Table 3:** Convergent and divergent (discriminant) validity of the PROMIS-A CAT *T*-scores.

PROM	rho	95% Confidence Interval for rho	*P*-value^a^
GAD-7	0.775	0.730–0.821	<0.001
ESAS-r anxiety	0.624	0.551–0.697	<0.001
EQ-5D-Anxiety/depression	0.668	0.601–0.734	<0.001
ESAS-r appetite	0.176	0.070–0.281	0.001
KCCQ-12 physical limitation	−0.270	(−0.377) to (−0.163)	<0.001
EQ-5D-Mobility	0.204	0.099–0.307	<0.001

For convergent validity, at least moderate correlation (rho > 0.6) was expected with legacy questionnaires measuring the same or similar construct (GAD-7, ESAS-r Anxiety, EQ-5D-Anxiety/Depression). For divergent (discriminant) validity, weak (rho < 0.4) correlation was expected between PROMIS-A CAT *T*-scores and scores measuring unrelated constructs (ESAS-r Appetite, KCCQ-Physical Limitation, EQ-5D-Mobility).

PROMIS-A, Patient-Reported Outcome Measurement Information System Anxiety; CAT, computer adaptive test; GAD-7, Generalized Anxiety Disorder-7; ESAS-r, Edmonton Symptom Assessment System Revised; EQ-5D, EuroQol 5-Dimension; KCCQ-12, Kansas City Cardiomyopathy Questionnaire-12; PROM, patient-reported outcome measure.

^a^ Alpha threshold for significance after Bonferroni correction for multiple tests = 0.01.

To further assess construct validity, PROMIS-A CAT and GAD-7 scores were analyzed across groups expected to have different anxiety levels (Table 4). PROMIS-A CAT *T*-scores and GAD-7 scores were both significantly higher among the youngest tertile of participants, and participants with ESAS-r scores ≥30, KCCQ-12 summary score <25, and PHQ-9 ≥10. Contrary to expectations, PROMIS-A CAT *T*-scores were not significantly different between male and female participants, comorbidity, or HF type. This pattern was similar for GAD-7 scores.

**Table 4:** Known-group comparisons for PROMIS-A CAT T-scores and GAD-7 scores—adjusted for age, sex, and EF. PROMIS-A CAT *T*-scores were adjusted in linear regression models using least-squared means function. GAD-7 scores were adjusted in quantile regression models; adjusted medians calculated by predict function. All adjustments performed in *R* version 4.3.3.

Known-group	PROMIS-A CAT *T*-score				GAD-7 score	
	Mean (SD)	*P*-value^a^	Cohen’s *d*	Median (IQR)	*P*-value^a^	Cliffs’ delta^b^
Age (years)^c^ *n* (%)						
19–62 119 (36)	58 (10)	<0.001		7 (11)	<0.001	
63–74 93 (28)	57 (11)			5 (8)		
75–98 121 (36)	51 (10)			2 (6)		
Sex^d^ *n* (%)						
Male 217 (65)	55 (10)	0.675	0.052	5 (7)	0.164	0.094
Female 116 (35)	56 (12)			6 (10)		
Charlson comorbidity index^e^ *n* (%)						
<4 288 (86)	55 (11)	0.727	0.054	5 (7)	0.189	0.126
≥4 45 (14)	56 (10)			6 (6)		
HF type^f^ *n* (%)						
HFrEF (LVEF < 40%) 171 (54)	55 (11)	0.631		4 (9)	0.105	
HFmEF (LVEF 40–49%) 47 (15)	56 (9)			6 (9)		
HFpEF (LVEF ≥ 50%) 101 (32)	55 (10)			5 (6)		
ESAS-r score^e^ *n* (%)						
<30 192 (58)	51 (10)	<0.001	0.929	3 (5)	<0.001	0.465
≥30 140 (42)	60 (9)			8 (9)		
KCCQ-12 summary score^e^ *n* (%)						
<25 109 (37)	60 (10)	<0.001	0.656	8 (10)	<0.001	0.403
≥25 182 (63)	53 (10)			4 (6)		
PHQ-9 Score^e^ *n* (%)						
<10 183 (55)	51 (10)	<0.001	0.945	3 (5)	<0.001	0.597
≥10 149 (45)	60 (9)			9 (9)		

PROMIS-A CAT, Patient-Reported Outcome Measurement Information System anxiety computer adaptive test; GAD-7, Generalized Anxiety Disorder-7; SD, standard deviation; IQR, interquartile range; HF, heart failure; LVEF, left ventricular ejection fraction; HFrEF, heart failure with reduced ejection fraction; HFmEF, heart failure with mid-range ejection fraction; HFpEF, heart failure with preserved ejection fraction; ESAS-r, Edmonton Symptom Assessment System Revised; KCCQ-12, Kansas City Cardiomyopathy Questionnaire-12; PHQ-9, Patient Health Questionnaire-9.

^a^ Alpha threshold for significance after Bonferroni correction for multiple tests = 0.0,035.

^b^ Effect size for differences in mean GAD-7 scores were determined using bootstrap resampling of 1,000 replications to account for skewed distribution of GAD-7 scores in sample.

^c^ Mean PROMIS-A CAT *T*-score/median GAD-7 score adjusted for sex and ejection fraction.

^d^ Mean PROMIS-A CAT *T*-score/median GAD-7 score adjusted for age and ejection fraction.

^e^ Mean PROMIS-A CAT *T*-score/median GAD-7 score adjusted for age, sex and ejection fraction.

^f^ Mean PROMIS-A CAT *T*-score/median GAD-7 score adjusted for age and sex.

**Figure 2:**
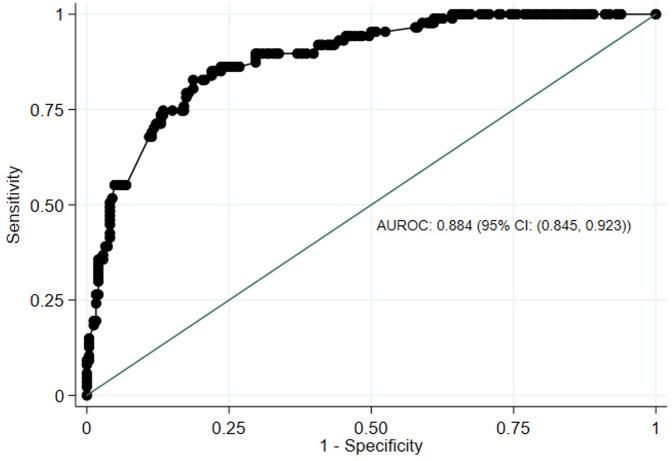
Receiver-operating characteristics (ROC) curve of PROMIS-anxiety CAT *T* scores against GAD-7 scores. AUROC, area under receiving-operating characteristics. PROMIS, Painted-Reported Income Measurement Information System; CAT, computer Adaptive Test; GAD-7, Generalized Anxiety Disorder-7; CI, Confidence Interval.

ROC curve analysis showed that PROMIS-A CAT *T*-scores had excellent discrimination between participants with vs. without moderate to severe anxiety based on GAD-7 ≥10 (AUROC: 0.885, 95% CI: 0.846–0.923) (Figure 2). Using Youden's J Index, PROMIS-A CAT *T*-scores of ≥59 or ≥60 were identified as potential thresholds for moderate to severe anxiety [sensitivity = 86% and 85%; specificity = 76% and 78%, respectively (Youden's *J* = 0.63 for both)] (Supplementary Table S1).

## 4 Discussion

This study provides evidence supporting the validity and reliability of the PROMIS-A CAT for detecting anxiety symptoms in patients hospitalized for HF. Our results demonstrated very good construct validity, high reliability, and excellent discrimination, which aligns with previous research across other patient populations (25, 62, 63). Given the excellent measurement characteristics, our results establish that PROMIS-A CAT can be considered for use in both clinical practice and research.

We first demonstrated validity of the PROMIS-A CAT when delivered to hospitalized patients with HF by determining its robust convergent validity. The strongest correlation was observed with GAD-7, which is expected since both are multi-item instruments measuring the same construct. The correlation between PROMIS-A (delivered by CAT or SF) and GAD-7 has been consistently strong in other studies (62–64). PROMIS-A CAT demonstrated moderate correlations with the ESAS-r anxiety item and EQ-5D anxiety/depression item. The ESAS-r anxiety assesses anxiety using only one item and has consequently shown only moderate correlation with the GAD-7 (34, 54, 65, 66). Similarly, EQ-5D assesses both anxiety and depression with a single item (38) and exhibits a significant floor effect, with only moderate correlation to other anxiety measures (67). Validity of PROMIS-A CAT *T*-score was further supported by weak correlation with scores assessing constructs unrelated to anxiety [divergent (discriminant) validity]. These correlation coefficients fall within the published range, supporting the robust validity of the PROMIS-A (49).

In known-group comparisons, PROMIS-A CAT *T*-scores differed between several, but not all, pre-specified sub-groups. Importantly, the distribution pattern was similar for GAD-7, as well, supporting construct validity. As expected, anxiety scores were higher among the youngest tertile of participants (ages: 19–62) vs. the middle (ages: 63–74) and oldest (ages: 75–98) tertiles; those with an ESAS-r score ≥30 (high symptom burden) vs. <30; those with a KCCQ-12 summary score <25 (poor HRQOL) vs. ≥25, and those with a PHQ-9 score ≥10 (moderate/severe depressive symptoms) vs. <10. Contrary to our hypotheses, both PROMIS-A CAT *T*-scores and GAD-7 scores were similar between female and male participants. However, similar findings have been reported (6, 68). It is possible that the disease severity or other sample characteristics contributed to this result. Similarly, no difference was detected between groups formed by comorbidity or HF type. Of note, those with non-ischemic cardiomyopathy made up a larger proportion of the moderate-severe anxiety cohort based on the GAD-7 ≥10. This can be attributed to younger average age among patients with this etiology, compared to coronary artery disease or ischemic etiologies that are more prevalent in older populations (69).

Our results showed excellent reliability of the PROMIS-A CAT, with individual-level reliability demonstrated by minimal standard error of measurements across the spectrum of PROMIS-A *T*-scores. This standard of high reliability is a built-in strength of PROMIS CAT, as standard stopping rules require a low standard error (theta <0.3) to stop additional testing (24, 25). Individual-level reliability decreased for participants with the least anxiety (lowest *T*-scores). This is not of clinical relevance, particularly if the tool is used for screening, as this score range falls well below the cut-off for potentially clinically significant symptom severity.

The PROMIS-A CAT exhibited excellent coverage across the range of anxiety severity, demonstrating no significant ceiling effect and a smaller floor effect compared to legacy measures. The floor effect for CAT administration in this study was lower than that observed when the item bank was administered as a SF in other patient populations (70–72), demonstrating a more tailored assessment with CAT (73).

PROMIS-A CAT demonstrated excellent discrimination between participants with vs. without moderate/severe anxiety. Our threshold analysis demonstrated near-identical specificity and sensitivity when using a cut-off score of 59 or 60, consistent with studies in other patient populations (64, 74). We recommend a cut-off *T*-score of ≥60 to identify patients with HF who may benefit from further assessment for potential moderate/severe anxiety. This threshold is one standard deviation above the reference value for the United States general population, which is congruent with the suggested distribution-based cut-off for moderate symptom burden across many PROMIS domains (75, 76).

Most participants in this study completed only 4–5 items with the PROMIS-A CAT to obtain a reliable score, compared to the seven required items with the GAD-7. This number of items is similar to findings in other patient populations completing PROMIS item banks via CAT (23, 77). On average, participants with no anxiety were required to answer more questions than those with higher *T*-scores. This is because these participants usually answered “never” to multiple items, which conveys insufficient information for the CAT to reach a low enough SEM to fulfill the stopping rule (78). This may elicit frustration; however, this can be addressed by modifying CAT stopping rules to limit the maximum number of items administered to 6 or 8, without losing precision. An even more efficient solution is a recent, optional modification, referred to as the “screen-to-CAT” method. If a participant selects “never” for the first item, no further question is asked (24, 79). Since the PROMIS items are calibrated based on the item response theory, even a single answer will yield a sufficiently reliable *T*-score.

Prior research has identified 7 core PROMIS domains (anxiety, depression, fatigue, pain interference, physical function, sleep disturbance, social functions), which greatly contribute to HRQOL (80, 81) and are relevant across many chronic conditions (82). Since PROMIS tools are not disease specific, they can be used to measure and compare these domains across many conditions (83). Based on average response times, it is possible to assess these 7 domains in 5–10 min, making PROMIS tools ideal candidates for symptom screening (25). Additionally, domain-specific *T*-scores can be combined to generate mental and physical health summary scores to characterize overall HRQOL on population level (84). Furthermore, PROMIS domain *T*-scores can be combined into a preference-based health utility score (PROMIS Preference score; PROPr), which provides an overall measure of patient quality of life and serves as a helpful metric for health economy analyses (85, 86).

PROMs and PROMIS tools are increasingly considered for clinical use, primarily for symptom assessment and monitoring. Major electronic medical record platforms now include PROM modules and PROMIS tools, allowing clinicians to efficiently track patient data and respond to changes in health status. This integration streamlines workflows, consolidates data, and enables patients to access their results through patient portals, empowering them to actively track and co-manage their own health parameters (87). A recent implementation trial showed feasibility when integrating PROMIS symptom scores into the electronic medical records of ambulatory oncology patients (88).

The results of this study should be interpreted in context of some limitations. We recruited a convenience sample of hospitalized patients with HF, which may not be representative of all hospitalized HF patients. Patients who felt more comfortable with electronics were likely overrepresented in this sample given the PROMIS-A CAT delivery method. Though, this should not impact the conclusions regarding validity and reliability of the PROMIS-A CAT *T*-scores. Nevertheless, computer literacy is an important consideration before implementation of CAT (25). Additionally, this analysis was cross-sectional and did not assess responsiveness of the PROMIS-A CAT; longitudinal validation is a focus for future work. Non-English speakers were excluded from recruitment; future studies should validate PROMIS-A CAT using translated item banks. Finally, while our study assessed the measurement characteristics of PROMIS-A CAT, the most efficient strategies for implementing the tool into clinical care remain unknown. Future implementation studies are needed to evaluate the feasibility of routine PROMIS-A CAT administration to screen and monitor for anxiety among patients with HF. Important considerations for these studies include assessing individual- and system-level barriers and facilitators toward adoption in the clinical context, understanding patient's and clinician's perceived utility of the instrument, and evaluating the impact of implementation on outcomes such as symptom burden, HRQOL, and healthcare use. It is important to note that in such studies, in addition to administering PROMIS-A CAT as a screening tool, it is essential for success that appropriate evidence-based symptom management pathways are available. Since the PROMIS tools are not diagnostic tools, individuals with potentially significant anxiety symptoms should be assessed by a qualified professional. If needed, resources for appropriate interventions, which may include, but are not limited to, self-care strategies, social support, psychotherapy, and medications, will need to be available (89).

## 5 Conclusion

In conclusion, we provide evidence supporting the reliability and validity of the PROMIS CAT anxiety item bank for hospitalized patients with HF. We recommend a cut-off score of ≥60 to identify patients with moderate/severe anxiety symptoms who may benefit from further clinical assessment. The PROMIS-A CAT demonstrates high measurement precision with fewer items than traditional legacy measures, making it an efficient tool for screening.

## Data Availability

The raw data supporting the conclusions of this article will be made available by the authors, without undue reservation.
