# Supplementary material for: Validation of PROMIS anxiety item bank computer adaptive test among patients with heart failure
Source: Front Cardiovasc Med. 2025 Oct 30;12:1605130. doi: 10.3389/fcvm.2025.1605130 (PMC12611916; doi:10.3389/fcvm.2025.1605130)
Supplement: Supplementary file 1 [file Supplementaryfile1.docx]

**Supplementary Figures & Tables**

**Table S1:** Threshold analysis of PROMIS Anxiety CAT compared with GAD-7 ≥ 10 for selected cut-off scores for PROMIS Anxiety CAT, for the entire cohort.

**Figure S1:** Flow chart of consenting participants to final sample size.

**Figure S2:** Histogram demonstrating the distribution of PROMIS-A-CAT T Scores.

**Figure S3:** Histogram demonstrating the distributions of (a) GAD-7, (b) ESAS-r Anxiety, (c) EQ-5D-Anxiety/Depression, and (d) ESAS-r Appetite overlaid with a normal distribution curve for the entire cohort.

**Figure S4:** Histogram demonstrating the distributions of (a) KCCQ-12 Physical Limitation, (b) KCCQ-12 summary score, (c) PHQ-9, and (d) EQ-5D-Mobility scores overlaid with a normal distribution curve for the entire cohort.

**Figure S5:** Linear fit plot (grey area corresponds to 95% CI) between PROMIS-A CAT T scores and (a) GAD-7 and (b) ESAS-r Anxiety.

**Figure S6:** Box plot between PROMIS-A-CAT T scores and EQ-5D-Anxiety/Depression.

**Figure S7:** Number of PROMIS-A-CAT items completed across the PROMIS-A-CAT T score spectrum for the entire cohort.

**Table S1:** Threshold analysis of PROMIS Anxiety CAT; reference: GAD-7 ≥ 10 (entire cohort).

| **Cut-off** | **Sensitivity** | **Specificity** | **TP** | **TN** | **FP** | **FN** | **PPV** | **NPV** | **Youden’s J** |
| --- | --- | --- | --- | --- | --- | --- | --- | --- | --- |
| 55 | 92% | 59% | 80 | 145 | 101 | 7 | 44% | 95% | 0.51 |
| 56 | 92% | 59% | 80 | 146 | 100 | 7 | 44% | 95% | 0.51 |
| 57 | 90% | 66% | 78 | 163 | 83 | 9 | 48% | 95% | 0.56 |
| 58 | 89% | 67% | 78 | 165 | 81 | 9 | 49% | 95% | 0.56 |
| **59** | **86%** | **76%** | **75** | **188** | **58** | **12** | **56%** | **94%** | **0.63** |
| **60** | **85%** | **78%** | **74** | **191** | **55** | **13** | **57%** | **94%** | **0.63** |
| 61 | 80% | 81% | 70 | 200 | 46 | 17 | 60% | 92% | 0.62 |
| 62 | 75% | 83% | 65 | 205 | 41 | 22 | 61% | 90% | 0.58 |
| 63 | 74% | 87% | 64 | 214 | 32 | 23 | 67% | 90% | 0.61 |
| 64 | 55% | 93% | 48 | 229 | 17 | 39 | 74% | 85% | 0.48 |
| 65 | 55% | 94% | 48 | 232 | 14 | 39 | 77% | 86% | 0.49 |

PROMIS, Patient-Reported Outcome Measurement Information System; CAT, computer adaptive test; GAD-7, Generalized Anxiety Disorder-7; TP, True positive; TN, True negative; FP, False positive; FN, False negative; PPV, Positive Predictive Value; NPV, Negative Predictive Value.

E

**Participants in PROBE-ML Study**

n = 520

**Participants dropped from final sample**

n = 187

Patients with missing clinical data: n = 19

Patients with missing legacy data (e.g. GAD-7, ESAS-r, EQ-5D-5L): n = 176

**Final sample size**

n = 333

**Figure S1:** Flow chart of consenting participants to final sample size. PROBE-ML, Predicting Readmission Outcomes using Biostatistical Evaluation and Machine Learning; GAD-7, Generalized Anxiety Disorder-7; ESAS-r, Edmonton Symptom Assessment System Revised; EQ-5D-5L, EuroQol 5-Dimension 5-Level.


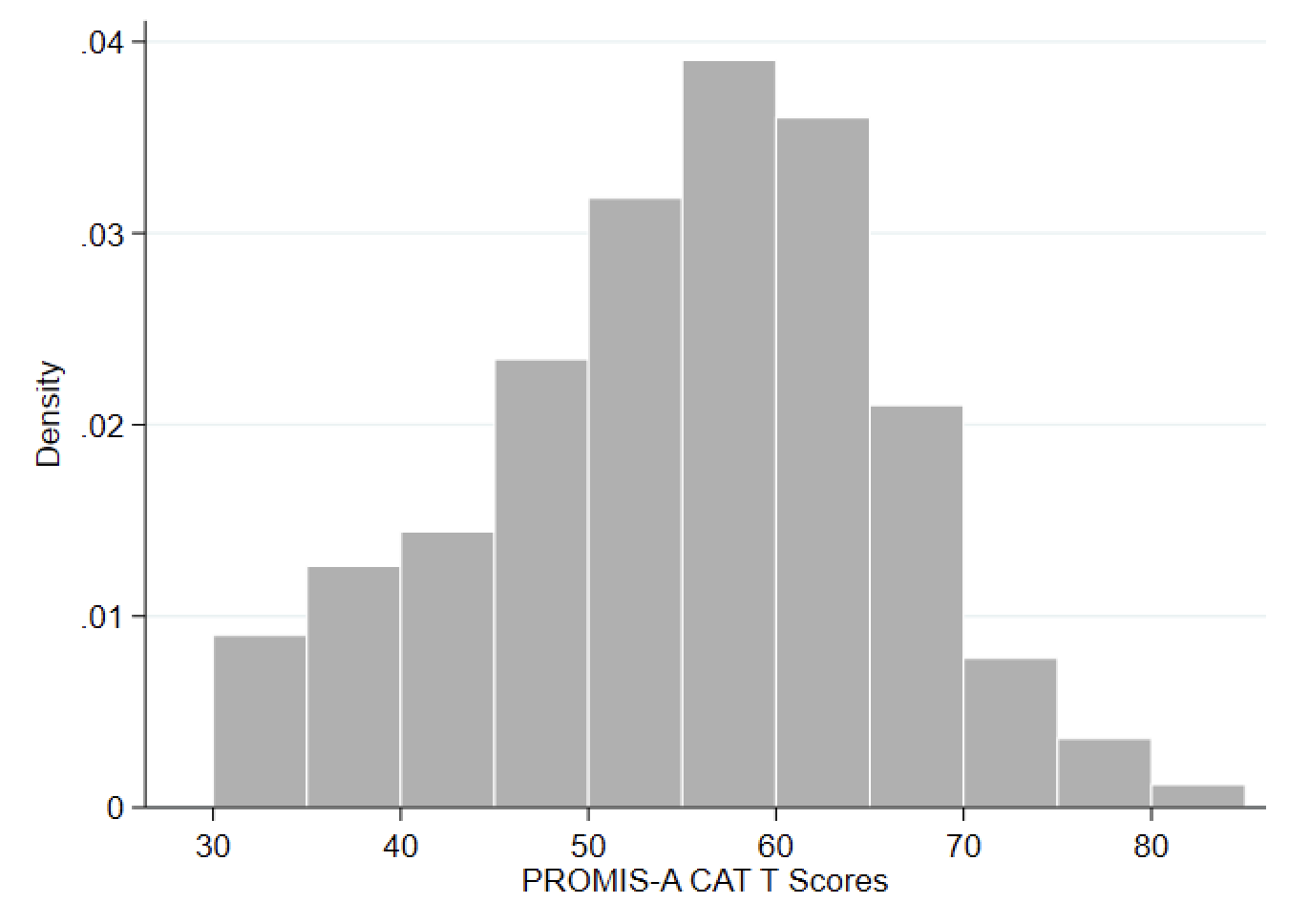


**Figure S2:** Distribution of PROMIS-A CAT T-scores. PROMIS-A CAT, Patient-Reported Outcomes Measures Information System Anxiety Computer Adaptive Test.

|  |  |
| --- | --- |
|  |  |

**
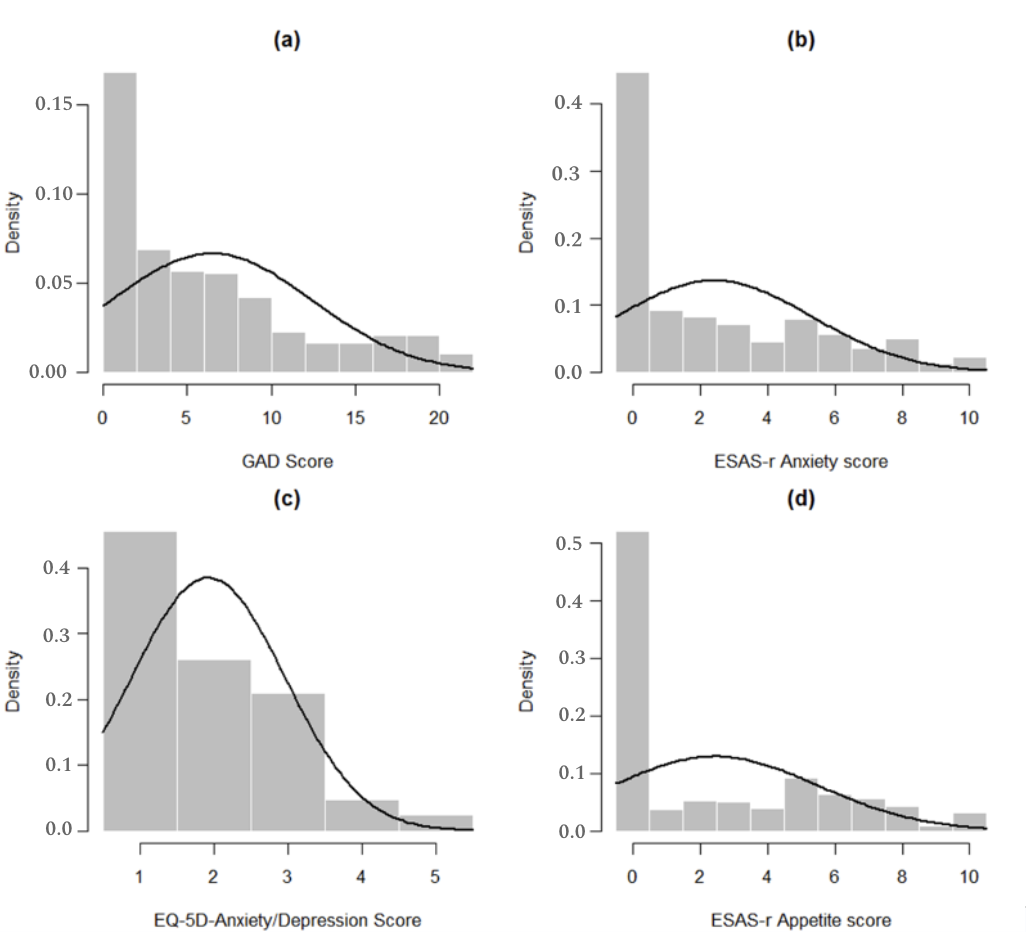
**

**Figure S3:** Distribution of (a) GAD-7 scores, (b) ESAS-r Anxiety scores, (c) EQ-5D-Anxiety/Depression scores and (d) ESAS-r Appetite scores. GAD-7, General Anxiety Disorder-7; ESAS-r, Edmonton Symptom Assessment System Revised; EQ-5D, EuroQol 5-Dimension.

**
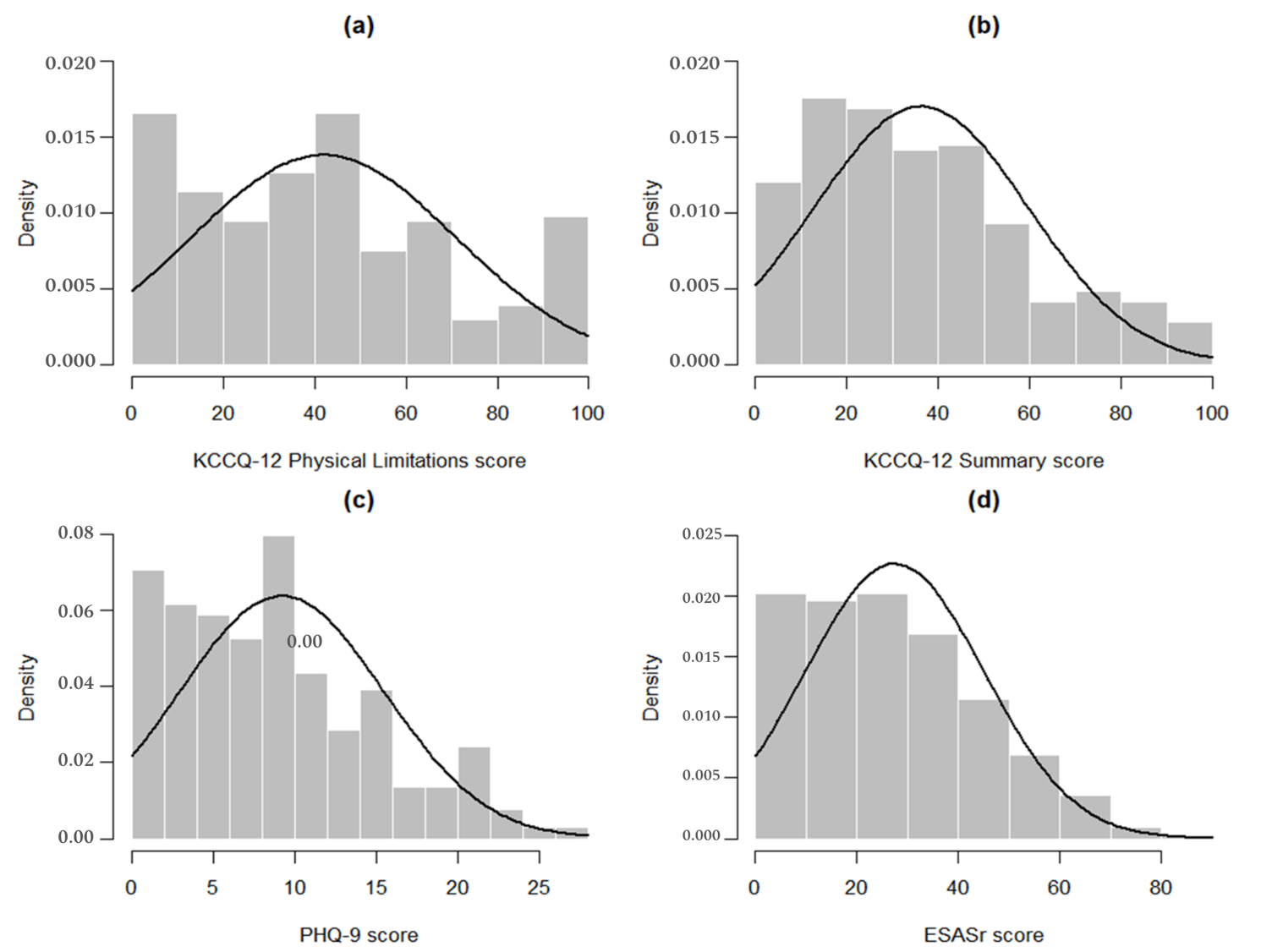
**

**Figure S4:** Distribution of (a) KCCQ-12 Physical Limitations scores, (b) KCCQ-12 Summary scores, (c) PHQ-9 scores and (d) ESAS-r scores. KCCQ-12, Kansas City Cardiomyopathy Questionnaire-12; PHQ-9, Patient Health Questionnaire-9; ESAS-r, Edmonton Symptom Assessment System Revised.


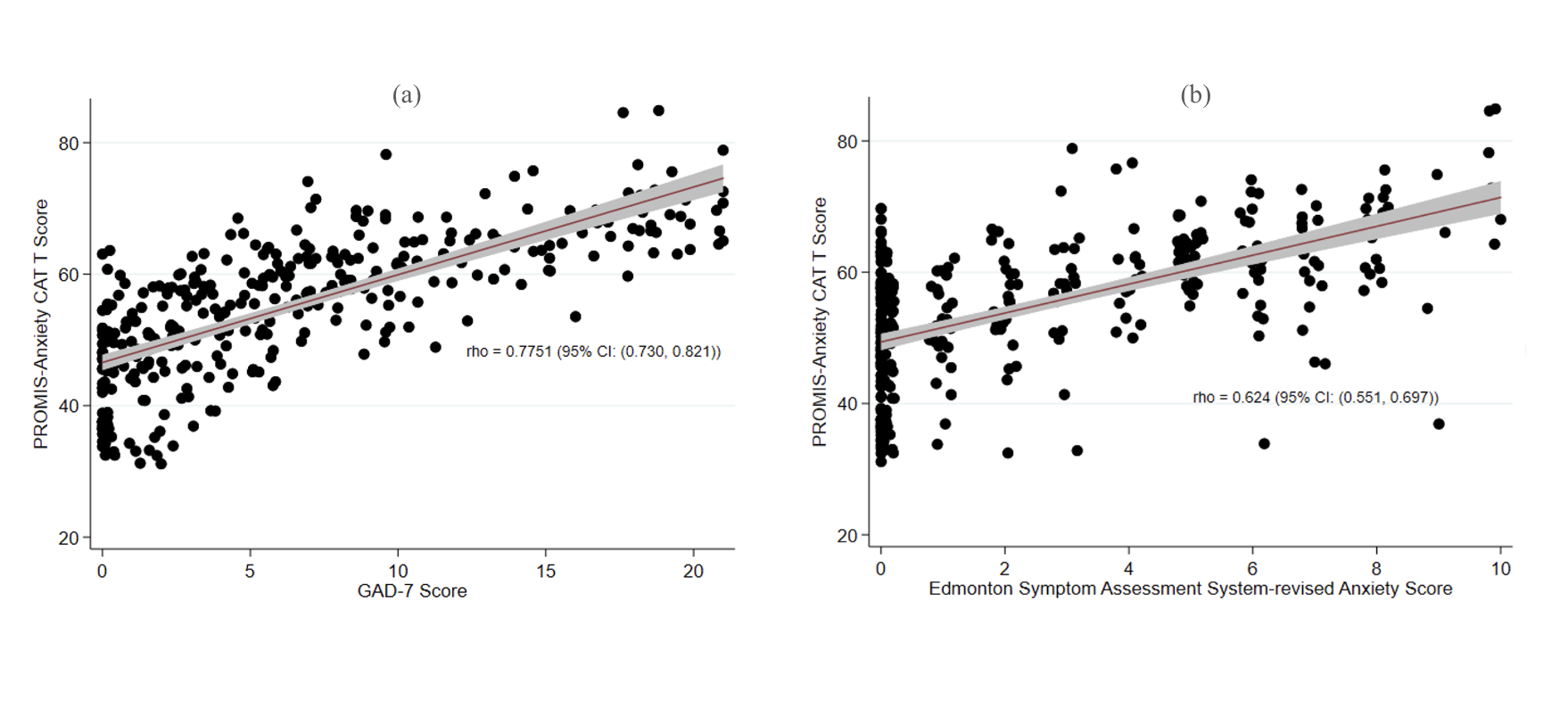


**Figure S5:** Linear fit plot [grey area corresponds to 95% CI] between PROMIS-A CAT T scores and (a) GAD-7 and (b) ESAS-r Anxiety. PROMIS, Patient-Reported Outcomes Measurement Information System; CAT, Computer Adaptive Test; GAD-7, Generalized Anxiety Disorder-7; ESAS-r, Edmonton Symptom Assessment System Revised; CI, confidence interval.

**
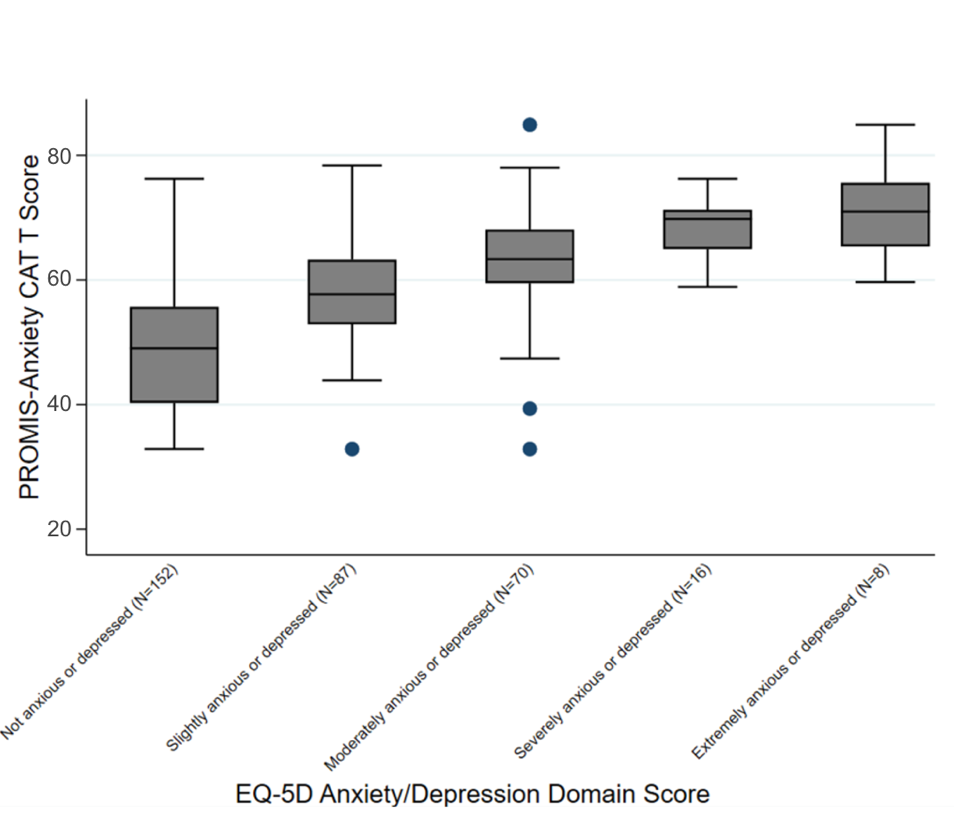
**

**Figure S6:** Box plot between PROMIS-A-CAT T scores and EQ-5D-Anxiety/Depression. PROMIS-A, Patient-Reported Outcomes Measurement Information System Anxiety; CAT, Computer Adaptive Test; EQ-5D, EuroQol 5-Dimension; CI, confidence interval.


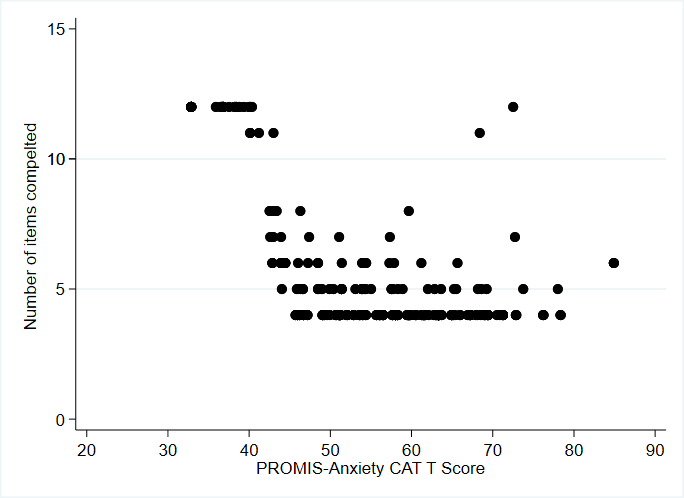


**Figure S7:** Number of PROMIS-A-CAT items completed across the PROMIS-A-CAT T score spectrum for the entire cohort. PROMIS-A, Patient-Reported Outcomes Measurement Information System Anxiety; CAT, Computer Adaptive Test.
